# Mitochondrial Alterations in Fibroblasts of Early Stage Bipolar Disorder Patients

**DOI:** 10.3390/biomedicines9050522

**Published:** 2021-05-07

**Authors:** Ana P. Marques, Rosa Resende, Diana F. Silva, Mariana Batista, Daniela Pereira, Brigite Wildenberg, Sofia Morais, António Macedo, Cláudia Pais, Joana B. Melo, Nuno Madeira, Cláudia F. Pereira

**Affiliations:** 1 Center for Innovative Biomedicine and Biotechnology (CIBB), University of Coimbra, 3004-504 Coimbra, Portugal; apatriciabmarques@gmail.com (A.P.M.); dianaffsilva@gmail.com (D.F.S.); mmelo@fmed.uc.pt (J.B.M.); 2 Center for Neuroscience and Cell Biology (CNC), University of Coimbra, 3004-504 Coimbra, Portugal; 3 Institute for Interdisciplinary Research (IIIUC), University of Coimbra, 3030-789 Coimbra, Portugal; 4 Clinical Academic Cener of Coimbra (CACC), 3004-561 Coimbra, Portugal; sousabatistamariana@gmail.com (M.B.); dsmpereira4@gmail.com (D.P.); brigitewildenberg@hotmail.com (B.W.); sofiamorais86@gmail.com (S.M.); amacedo@ci.uc.pt (A.M.); nmadeira@uc.pt (N.M.); 5 Department of Dermatology, Centro Hospitalar e Universitário de Coimbra (CHUC), 3004-561 Coimbra, Portugal; 6 Faculty of Medicine, University of Coimbra, 3000-548 Coimbra, Portugal; 7 Department of Psychiatry, Centro Hospitalar e Universitário de Coimbra (CHUC), 3004-561 Coimbra, Portugal; 8 Institute for Biomedical Imaging and Translational Research (CIBIT), University of Coimbra, 3000-548 Coimbra, Portugal; 9 Cytogenetics and Genomics Laboratory, Faculty of Medicine, University of Coimbra, 3000-548 Coimbra, Portugal; claudypais@gmail.com; 10 Coimbra Institute for Clinical and Biomedical Research (iCBR), Center of Investigation in Environment, Genetics and Oncobiology (CIMAGO), 3000-548 Coimbra, Portugal

**Keywords:** bipolar disorder, mitochondrial dysfunction, mitochondrial biogenesis, mitophagy, bioenergetics, fibroblasts

## Abstract

This study aims to evaluate whether mitochondrial changes occur in the early stages of bipolar disorder (BD). Using fibroblasts derived from BD patients and matched controls, the levels of proteins involved in mitochondrial biogenesis and dynamics (fission and fusion) were evaluated by Western Blot analysis. Mitochondrial membrane potential (MMP) was studied using the fluorescent probe TMRE. Mitochondrial morphology was analyzed with the probe Mitotracker Green and mitophagy was evaluated by quantifying the co-localization of HSP60 (mitochondria marker) and LC3B (autophagosome marker) by immunofluorescence. Furthermore, the activity of the mitochondrial respiratory chain and the glycolytic capacity of controls and BD patients-derived cells were also studied using the Seahorse technology. BD patient-derived fibroblasts exhibit fragmented mitochondria concomitantly with changes in mitochondrial dynamics and biogenesis in comparison with controls. Moreover, a decrease in the MMP and increased mitophagy was observed in fibroblasts obtained from BD patients when compared with control cells. Impaired energetic metabolism due to inhibition of the mitochondrial electron transport chain (ETC) and subsequent ATP depletion, associated with glycolysis stimulation, was also a feature of BD fibroblasts. Overall, these results support the fact that mitochondrial disturbance is an early event implicated in BD pathophysiology that might trigger neuronal changes and modification of brain circuitry.

## 1. Introduction

Bipolar disorder (BD) is a chronic and debilitating mental disorder that affects about 45 million people worldwide [1] and is characterized by frequent changes in mood, between mania/hypomania and depression, expressed as recurrent changes in energy levels and behavior [2]. Type I BD is diagnosed on the basis of ≥1 lifetime occurrence of mania, while Type II is characterized by episodes of depression and hypomania [2]. This pathology usually manifests in adolescence or early adulthood, with the mean age of onset being 20 years old, and remains throughout life [3]. This psychiatric illness is considered by the World Health Organization to be the fourth leading cause of disability in individuals aged between 15 and 44 years [4].

To avoid recurrences of mood episodes in BD, lifetime treatment is frequently needed and is essentially pharmacological, using mood stabilizers (e.g., lithium and valproate) or atypical antipsychotics (e.g., quetiapine and aripiprazole); antidepressants are also useful for acute depressive phases [2]. Notwithstanding advances in neuroscientific research, no clinical or laboratorial biomarker has been clearly identified that allows for an early and accurate diagnosis of BD [5,6]. Thus, a better knowledge of the underlying mechanisms of BD pathophysiology is still an unmet need. Delayed diagnosis/misdiagnosis is frequent, especially in early phases of BD, given that its onset is often characterized by a depressive episode, which can be classified as unipolar depression [7]. It has been suggested that cellular and molecular alterations that can modify neuronal interconnectivity play a role in the pathophysiology of BD, including mitochondrial dysfunction, endoplasmic reticulum (ER) stress, neuroinflammation, redox alterations, epigenetic changes and apoptosis [8,9,10].

Much evidence from genetic, functional and neuroimaging studies, suggesting that mitochondrial dysfunction may play a key role in BD pathophysiology, has been published in the last two decades [8,11]. Interestingly, primary mitochondrial diseases increase susceptibility to BD [12].

Mitochondria are highly dynamic organelles that produce ATP through oxidative phosphorylation via electron transport chain (ETC) that establish the equilibrium between health and disease. Adaptation of energy supply to energy needs is crucial to maintain cellular bioenergetics, which is regulated by the mitochondrial dynamics and turnover [13].

Magnetic resonance spectroscopy (MRS) studies have shown alterations in neurometabolites that are hallmarks of decreased mitochondrial energy production [11]. Furthermore, increased levels of lactate and gamma-aminobutyric acid have been detected in the gray matter of medication-free BD patients, suggesting a shift from oxidative phosphorylation toward glycolysis in BD [14]. Decreased expression of many messenger RNAs (mRNAs) encoding subunits of the ETC complexes I to V has been described in the hippocampus [15] and in the prefrontal cortex from BD patients [16,17]. Defects in the ETC result in increased production of reactive oxygen species (ROS), leading to oxidative stress [18]. In fact, markers of oxidative damage, namely lipid peroxidation, DNA/RNA damage, and nitric oxide were found to be increased in BD patients [11]. In addition to alterations in energy metabolism there are numerous studies focusing the role in BD of the mitochondrial quality control system which operates through the coordination of various processes (proteostasis, biogenesis, dynamics, and mitophagy) to ensure cell homeostasis [19]. Previous research findings have reported alterations in mitochondrial morphology in postmortem prefrontal cortical neurons and in neurons derived from induced pluripotent stem cells (iPSCs) of patients with BD [20,21]. More recently, Scaini et al. described an imbalance in mitochondrial fission and fusion and an impaired mitophagy pathway in peripheral blood mononuclear cells (PBMCs) from BD patients [22,23].

In this study, we evaluated mitochondrial alterations in fibroblasts of BD patients. According to our results, BD patient-derived fibroblasts exhibit fragmented mitochondria concomitantly with changes in mitochondrial dynamics and biogenesis in comparison with controls. Moreover, we observed a decrease in the mitochondrial membrane potential and increased mitophagy in fibroblasts obtained from BD patients when compared with control cells. Finally, impaired energetic metabolism due to inhibition of mitochondrial ETC and subsequent ATP depletion, associated with glycolysis stimulation, was found in BD fibroblasts in comparison with controls. Overall, these results support the fact that mitochondrial disturbance is an early event implicated in BD pathophysiology that might trigger neuronal changes and the modification of brain circuitry.

## 2. Experimental Section

### 2.1. Patients and Fibroblasts Cell Culture

Skin fibroblasts were obtained from five patients with BD in early phases—BD stage 2 [24], with ages between 18 and 35 years-old, and five age- and gender-matched healthy controls. Patients were assessed regarding a DSM-5 [25] BD diagnosis through a validated diagnostic interview [26]. Controls were selected among students and health professionals from our University. Informed consent was provided to all patients and controls, and the study was approved by the Ethics Committee of the Centro Hospitalar e Universitário de Coimbra (150/CES, July 3rd).

Dermal fibroblasts were generated from skin biopsies (3 mm) performed in subjects who agreed to participate, having no contraindications for cutaneous biopsy (e.g., coagulation problems), and after informed consent in accordance with local Ethics Committee’s guidelines. After 1–2 weeks in culture, fibroblast outgrowths were sub-cultured and expanded as previously described [27]. Characteristic spindle-shape morphology was confirmed by optical microscopy.

Fibroblasts were maintained in HAM’s F10 (#31550-023 ThermoFisher Scientific, Waltham, MA, USA) medium supplemented with 10% (*v*/*v*) heat inactivated fetal bovine serum (FBS), 1% (*v/v*) antibiotic solution (10,000 U/mL penicillin, 10,000 μg/mL streptomycin) and 1% (*v/v*) L-Glutamine and AmnioMAX™-II Complete Medium (#11269-016, ThermoFisher Scientific) in a proportion of 1:5. Cells were cultured in 75 cm^2^ flasks and maintained in a humidified 5% CO_2_-95% air atmosphere at 37 °C. The medium was changed every 3 days. Cultures were passaged by trypsinization when cells reached 70–80% confluence. The fibroblasts used in the experiments had between 7 and 15 passages.

### 2.2. Mitochondria Morphology Analysis

Cells were seeded in 12-well plates with 18 mm coverslips at a density of 0.1 × 10^5^ cells/cm^2^, 48 h after plating, cells were washed three times in Krebs medium (140 mM NaCl, 5 mM KCl, 1.2 mM Na_2_HPO_4_, 1 mM MgCl_2_, 9.6 mM Glucose, 20 mM Hepes, 1 mM CaCl_2_, pH 7.4) and incubated with 0.1 µM Mitotracker Green (#m7514 Invitrogen) for 30 min at 37 °C. Then, cells were incubated with 15 µg/mL Hoechst 33342 during 5 min to stain the nuclei. Confocal images were obtained using a Zeiss LSM 710 confocal microscope (Zeiss Microscopy, Germany). The experiments were carried out in duplicate and 5 images were analyzed.

#### Image Processing

Mitochondrial morphology was analyzed using Macros designed in Fiji (ImageJ, National Institute of Health, Bethesda, MA, USA) by Dr. Jorge Valero (CNC, University of Coimbra, presently at Achucarro—Basque Centre for Neuroscience, Spain) as described by Naia et al. [28]. We obtained the roundness, which is a two-dimensional index of mitochondrial sphericity (the relation between mitochondrial area and its major axis), aspect ratio that measures length (the ratio between the major and minor axis of mitochondria), as well as form factor, which is a measure of the degree of mitochondria branching (FF = Perimeter2/4π × area).

### 2.3. Immunocytochemistry

Cells were fixed with 4% (*w/v*) paraformaldehyde for 15 min, permeabilized with 0.1% (*v/v*) Triton X-100 for 5 min and blocked with 3% (*w/v*) BSA prepared in 0.2% (*v/v*) Tween 20, for 1 h at room temperature (RT). Cells were then incubated with the antibodies mouse anti-HSP60 (1:1000 #mab3844 Chemicon, Temecula, CA, USA) and rabbit anti-LC3B (1:1000; #2775 Cell Signaling Technology, Danvers, MA, USA) overnight at 4 °C, washed three times and then incubated with the secondary antibodies at RT for 1 h. At last, cells were incubated for 5 min with Hoechst 33342 (15 μg/mL) and the coverslips were mounted using Aqua-Poly/Mount mounting medium (#18606 Polysciences Inc., Warrington, PA, USA). Confocal images were obtained using a Zeiss LSM 710 confocal microscope (Zeiss Microscopy, Germany). The fraction of autophagosomes (LC3B) that co-localize with mitochondria (HSP60) and the fraction of mitochondria that co-localize with autophagosomes was measured with Mander’s coefficient of co-localization using JACoP plugin in Fiji (ImageJ, National Institute of Health, Bethesda, MA, USA) [29]. The experiments were carried out in duplicate and 5 images were analyzed.

### 2.4. Mitochondrial Membrane Potential

Mitochondrial membrane potential (MMP) was assessed using the tetramethylrhodamine ester (TMRE) probe (#87917 Sigma-Aldrich, St. Louis, MO, USA). Human fibroblasts were seeded in 96-well plates for 48 h at 37 °C and 5% CO_2_-95% air, at a density of 0.3 × 10^5^ cells/cm^2^. Then, the cell culture medium was replaced by Krebs medium containing TMRE (1 μM) and cells were incubated for 30 min at 37 °C and 5% CO_2_-95% air. The fluorescence was read at 540 (excitation) and 595 nm (emission) wavelengths in a microplate reader (SpectraMax Gemini EM fluorocytometer, Molecular Devices, CA, USA).

### 2.5. Protein Isolation and Western Blot Analysis

Fibroblasts were lysed in RIPA buffer [250 mM NaCl, 50 mM Tris base, 1% (*v/v*) Nonidet P-40, 0.5% (*v/v*) sodium deoxycholate (DOC), 0.1% (*v/v*) Sodium Dodecyl Sulfate (SDS), pH 8.0] supplemented with 2 mM DTT, 1% (*v/v*) of the protease inhibitor cocktail (#P2714 Sigma), 100 μM PMSF, 2 mM sodium ortovanadate and 50 mM sodium fluoride. After 20 min on ice, cell lysates were centrifuged at 17,968× *g* for 10 min at 4 °C and the supernatants were collected and stored at −20 °C.

Western blot (WB) analysis was performed as previously described [30,31]. Briefly, thirty micrograms from total cell lysates were separated by electrophoresis in 10–15% SDS polyacrylamide gels (SDS/PAGE) and transferred to PVDF membranes (#ipvh00010 Merck Millipore, Burlington, MA, USA). After blocking the membranes were incubated overnight at 4 °C with the following primary antibodies: rabbit anti-Fis1 (1:1000; #NB100-56646 Novus Biologicals, Littleton, CO, USA), goat anti-mtTFA (A-17) (1:1,000; #sc-23588 Santa Cruz Biotechnology, Inc., Dallas, TX, USA), rabbit anti-PGC1-α (1:500; # sc-13067 Santa Cruz Biotechnology, Inc.), mouse anti-MFN2 (1:1,000; #sc-100560 Santa Cruz Biotechnology, Inc.), rabbit anti-MFN1 (1:500; #sc-50330 Santa Cruz Biotechnology, Inc.), rabbit anti-SQSTM1/p62 (1:1000; #5114 Cell Signaling Technology, Danvers, MA, USA), mouse anti-OPA1 (1:1000; #612606 BD Transduction Laboratories, Franklin Lakes, NJ, USA), rabbit anti-pDRP (Ser616) 1:500; #1673455S Cell Signaling Technology), rabbit anti-DRP (D6C7) (1:1000; #1678570S Cell Signaling Technology) and mouse anti-Parkin (Prk8) (1:1,000; #4211 Cell Signaling Technology). After six washes with TBS-T, the membranes were incubated for 1 h, at RT, with a peroxidase conjugated goat anti-mouse IgG (1:20,000 or 1:50,000 #31432 ThermoFisher Scientific, Waltham, MA, USA), goat anti-rabbit IgG (1:20,000; #31462 ThermoFisher Scientific) or rabbit anti-goat IgG (1:20,000; #31402 ThermoFisher Scientific). The protein immunoreactive bands were visualized by chemiluminescence with the Pierce ECL Western Blotting Substrate (#32106 ThermoFisher Scientific) in a Chemidoc Imaging System (Bio-Rad Laboratories, Lda, Algés, Portugal). The monoclonal anti-β-actin antibody (1:10,000; #A5441 Sigma) was used for protein loading control. The optical density of the bands was quantified with the Image Lab Software (Bio-Rad).

### 2.6. Seahorse XF24 Extracellular Flux Analyzer Measurements

Mitochondrial oxygen consumption rate (OCR) from fibroblasts from 5 controls and 3 BP was evaluated by seeding approximately 3.5 × 10^4^ cells per well in 24 well cell culture microplates provided by the manufacturer (#100777-004 Agilent, Santa Clara, CA, USA). The day after seeding and 1 h before placing culture microplates in the Seahorse Analyzer, the cells were washed in unbuffered DMEM (#5030 Sigma Chemical Co., St. Louis, MO, USA) without HEPES and sodium bicarbonate. The medium was then changed to unbuffered DMEM containing 25 mM glucose, 5 mM sodium pyruvate and 2 mM L-glutamine, at pH 7.4. Measurement of OCR was done at baseline and following sequential injections of (i) oligomycin (1 μM), an ATP synthase inhibitor, (ii) carbonyl cyanide-4-(trifluoromethoxy) phenyl hydrazone (FCCP) (7 μM), a mitochondrial uncoupler, and (iii) a mixture of rotenone (2 μM) and antimycin A (2 μM), which are complex I and complex III inhibitors, respectively. The first OCR value after rotenone and antimycin injection provides the non-mitochondrial respiration and was used to correct the previously described OCRs.

For glycolysis analysis, fibroblasts were washed on the assay day in unbuffered DMEM and then incubated for 1 h at 37 °C in unbuffered glucose-free DMEM supplemented with 2 mM L-glutamine and 5 mM sodium pyruvate. After incubation for 1 h in a non-CO_2_ incubator at 37 °C, cells were transferred to the Seahorse Analyzer and extracellular acidification rate (ECAR) measurements were done at baseline and following sequential injections of (i) glucose (25 mM), (ii) oligomycin (1 µM). Finally, an injection of 2-deoxyglucose (100 mM), a competitive inhibitor of hexokinase, provided a non-glycolysis ECAR. The lowest ECAR value after 2-deoxyglucose injection provides the non-glycolytic acidification rate and was used to correct ECAR value after oligomycin injection.

### 2.7. Data Analysis

Statistical analysis was performed using the Mann–Whitney non-parametric test. A value of *p* < 0.05 was considered significant. Prism (GraphPad Software version 6.0, San Diego, CA, USA) was used for all statistical analysis and the results were expressed as mean ± SEM. Given the small number of samples, statistical power was limited. To increase power, technical replicate/image was considered independently in the seahorse and microscopy experiments. To avoid over-sampling of any particular donor, the number of replicates and images was balanced in each sample.

## 3. Results

### 3.1. Mitochondrial Morphology Is Altered in BD Patients-Derived Fibroblasts

**Figure 1:**
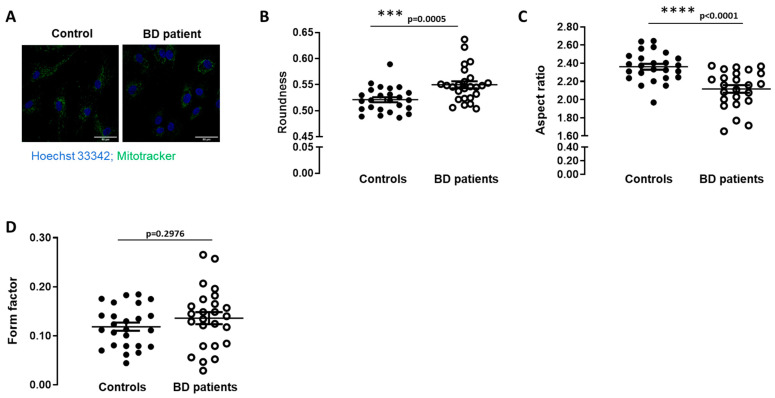
Mitochondrial morphology in BD patients’ fibroblasts. Mitochondrial morphology was analyzed by fluorescent microscopy using Mitotracker Green. (**A**) Representative confocal microscopy images of the mitochondrial network shown in green and Hoechst 33342-stained nuclei in blue (magnification, ×40). (**B**) Mitochondrial roundness, (**C**) mitochondrial aspect ratio, (**D**) mitochondrial form factor were determined through the analysis of the fluorescence of confocal microscopy images. Data are the mean ± SEM of 5 different individuals of each group. The experiments were carried out in duplicate and 5 images were analyzed. *** *p* < 0.001; **** *p* < 0.0001, significantly different from control group, as determined by Mann–Whitney non-parametric test.

Several studies suggest that BD may be associated with alterations in mitochondrial function, so mitochondrial morphology changes were evaluated by immunocytochemistry in fibroblasts obtained from BD patients and matched controls. Significant differences in mitochondrial morphology were found in BD patients’ fibroblasts compared to controls, namely disruption of the mitochondrial network (Figure 1). Quantitative morphological analysis was performed using ImageJ, obtaining three variables: Roundness (correlated with the degree of circularity), Aspect Ratio (positively correlated with length), and Form Factor (measure of the degree of mitochondrial branching). Our results showed decreased mitochondrial length and increased roundness in BD fibroblasts, without a significant change in mitochondrial branching (Figure 1). These findings demonstrate altered mitochondrial morphology suggesting disruption of mitochondrial network in BD fibroblasts during early stages of the disease.

### 3.2. Balance between Fission and Fusion Is Disrupted in BD Patients-Derived Fibroblasts

**Figure 2:**
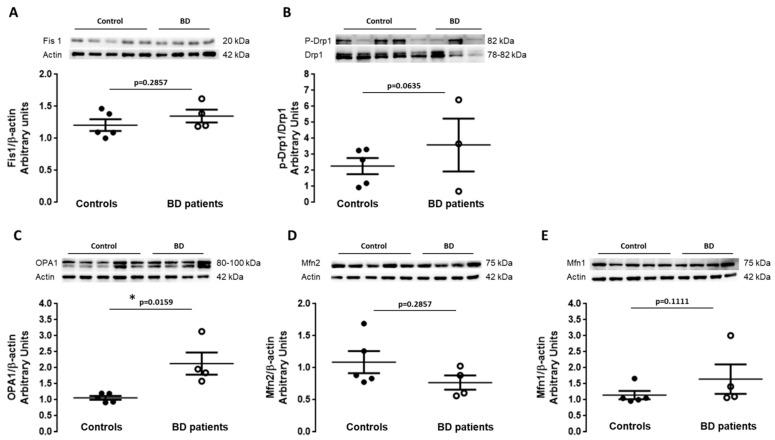
Fission/fusion machinery in BD patients’ fibroblasts. The levels of the mitochondrial fission proteins (**A**) Fis1 and (**B**) p-Drp1 and the mitochondrial fusion proteins (**C**) OPA1 (**D**) Mfn2 and (**E**) Mfn1, in fibroblasts from BD patients and control individuals were evaluated by Western blot analysis. Drp1 was used as the loading control for p-Drp1 (S616) and β-actin was used as loading control for the other proteins. The results were normalized to β-actin or Drp1 and expressed as mean ± SEM of 5 different control individuals and 3-4 BD patients. WB representative images correspond to BD and control samples analyzed on the same gel. * *p* < 0.05, significantly different from control group, as determined by Mann–Whitney non-parametric test.

Mitochondria are highly dynamic organelles acting as a point of equilibrium between health and disease. The adaptation of energy supply to energy demand is crucial to cellular bioenergetic homeostasis and is critically regulated by fission and fusion processes [32,33]. Since we observed alterations in mitochondria morphology suggesting mitochondria fragmentation, the levels of proteins involved in both fission and fusion were analyzed in cell lysates of control- and BD patient-derived fibroblasts. To investigate whether mitochondrial fission was altered, the protein levels of mitochondrial fission 1 (Fis1) and dynamin-related protein 1 (Drp1) were analyzed by WB. Changes in the protein levels of Mitofusin 1 (Mfn1), Mitofusin 2 (Mfn2) and optic atrophy 1 (OPA1), which are markers of mitochondrial fusion, were also determined by WB. Protein levels of Fis1 and p-Drp1 are slightly increased in fibroblasts obtained from BD patients when compared with control cells. Moreover, we observed a significant increase in OPA1 levels, a tendency to increase in Mfn1 and decrease in Mfn2 levels in BD patient-derived fibroblasts (Figure 2A–E). These data suggest an imbalance between mitochondrial fission and fusion in BD fibroblasts.

### 3.3. Mitochondrial Biogenesis Is Increased in BD Patients-Derived Fibroblasts

**Figure 3:**
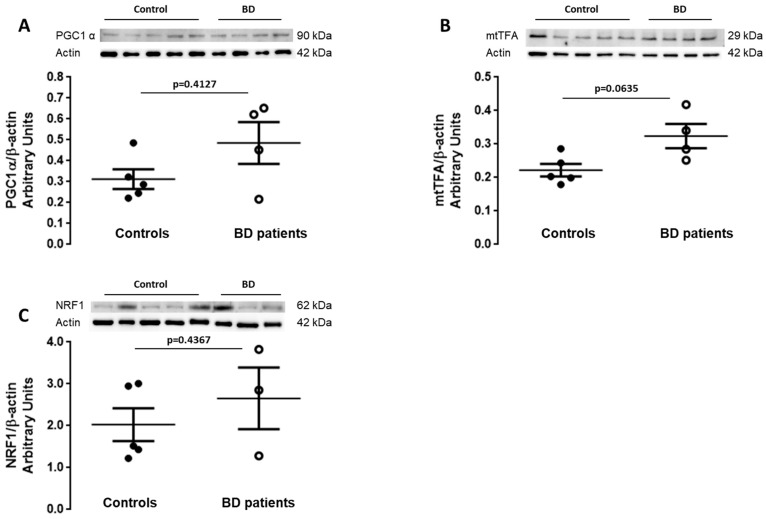
Mitochondrial biogenesis in BD patients’ fibroblasts. The protein levels of mediators of mitochondrial biogenesis (**A**) PGC1α (**B**), mtTFA and (**C**) NRF1 were evaluated in fibroblasts from BD patients and control individuals through Western blot analysis. β-actin was used as loading control. The results were normalized to β-actin and expressed as mean ± SEM of 5 different control individuals and 4 BD patients. WB representative images correspond to BD and control samples analyzed on the same gel. A Mann–Whitney non-parametric test was used to statistical analysis.

Mitochondrial biogenesis is an important process for regulating the number of mitochondria in the cell. Therefore, we studied the levels of peroxisome proliferator-activated receptor γ coactivator-1 (PGC1α), mitochondrial transcription factor 1 (mtTFA) and nuclear respiratory factor 1 (NRF1) that play important roles in mitochondrial biogenesis [34]. Our results show that BD patient-derived fibroblasts exhibit a tendency for an increase in the levels of PGC-1α, mtTFA (*p* = 0.0635) and NRF1 that did not reach statistical significance. However, taking into account the small number of samples, we consider the tendencies relevant. An increase in mitochondrial biogenesis in BD patients might be a cellular response in order to compensate mitochondrial impairment (Figure 3A–C).

### 3.4. Macroautophagy and Mitophagy Are Stimulated in BD Patients-Derived Fibroblasts

**Figure 4:**
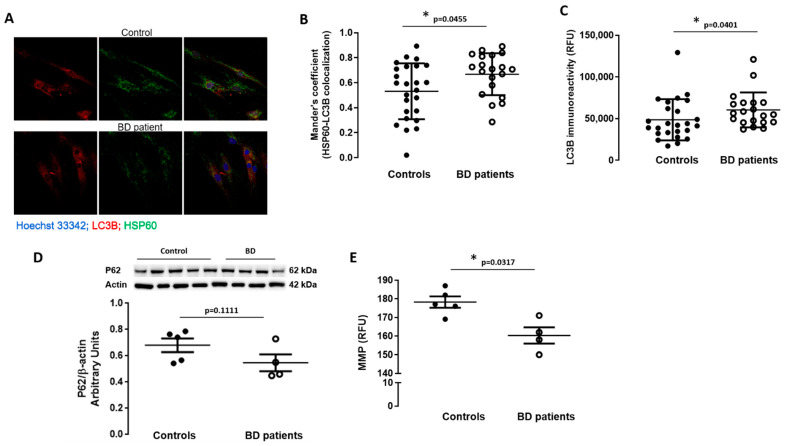
Mitophagy-associated proteins in BD patients’ fibroblasts. Co-localization of LC3B (autophagosome marker) and HSP60 (mitochondrial marker) was evaluated by immunocytochemistry. (**A**) Representative confocal microscopy images of LC3B (red) and HSP60 (green) immunoreactivity and nuclei labelling with Hoechst 33,342 (blue) (magnification, ×40). LC3B and HSP60 co-localization (**B**) as well as LC3B staining (**C**) were quantified using the ImageJ software. The experiments were carried out in duplicate and 5 images were analyzed. The protein levels of p62 (**D**) were evaluated in fibroblasts from BD patients and control individuals through Western blot analysis. β-actin was used as loading control. The results were normalized to β-actin. The mitochondrial membrane potential (**E**) was assessed using the fluorescent probe TMRE. Basal fluorescence (excitation: 505 nm; emission: 525 nm) was measured using a microplate reader. Data are expressed as mean ± SEM of 5 different control individuals and 4 BD patients. WB representative images correspond to BD and control samples analyzed on the same gel. * *p* < 0.05, significantly different from control group, as determined by Mann–Whitney non-parametric test.

Autophagy is a process by which intracellular components are degraded in the lysosome. This can occur by three types of autophagy: microautophagy, chaperone-mediated autophagy, and macroautophagy. Mitophagy is a form of macroautophagy, which degrades dysfunctional mitochondria upon their engulfment in the autophagosome that in turn fuses with the lysosome, thus maintaining cellular homeostasis and protecting cells against the accumulation of damaged mitochondria [35]. Therefore, to better understand the role of mitochondria in BD, mitophagy was studied by assessing co-localization of mitochondria (Hsp60) with autophagosomes (LC3B) (Figure 4A,B). We observed an increase in the degree of Hsp60 and LC3B co-localization in fibroblasts from BD patients in comparison with controls (Figure 4B). We also evaluated protein levels of the autophagy-associated proteins LC3B and p62 (macroautophagy substrate) by immunofluorescence and WB, respectively (Figure 4C,D). Increased LC3B staining together with slight p62 depletion was observed in BD patients’ fibroblasts compared with controls, suggesting induction of macroautophagy in the former cells (Figure 4). Taken together, these results suggest activation of macroautophagy in an attempt to eliminate dysfunctional mitochondria in early BD.

### 3.5. Mitochondrial Membrane Depolarization in BD Patients-Derived Fibroblasts

The ability of mitochondria to perform their functions greatly depends on the maintenance of the mitochondrial membrane potential (MMP) as depolarized mitochondria exhibit altered mitochondrial dynamics. Mitochondrial fission is necessary for mitochondrial degradation by mitophagy because fission enables the separation of depolarized mitochondria from the mitochondrial network and allows their engulfment by autophagosomes [10].

The degree of mitochondrial membrane polarization was assessed and we observed a significant decrease in MMP in fibroblasts obtained from BD patients in comparison with fibroblasts derived from controls (Figure 4E).

### 3.6. Mitochondrial Respiration and Glycolytic Capacity in BD Patients-Derived Fibroblasts

**Figure 5:**
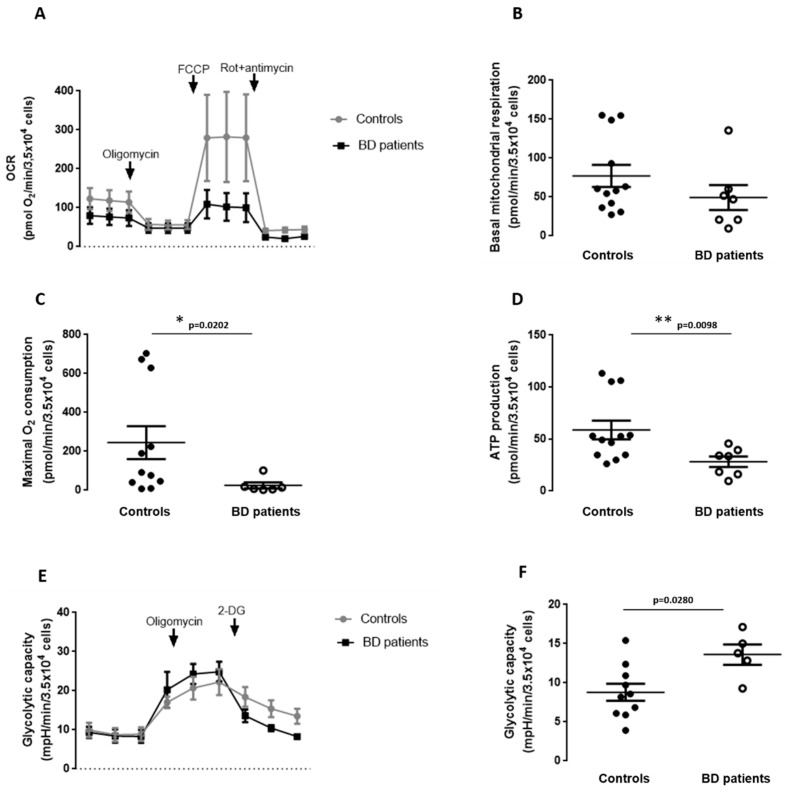
Mitochondrial respiration and glycolytic capacity in BD patients’ fibroblasts. Oxygen consumption rate (OCR) was evaluated in fibroblasts from BD patients and age-matched controls. (**A**–**F**) Mitochondrial respiration was assessed by sequential addition of oligomycin, FCCP and rotenone/antimycin A by using a Seahorse XF24 flux analyzer. (**A**) Representative traces depicting the average of *n* = 5 controls and *n* = 3 BD patients-derived fibroblasts. (**B**) Basal mitochondrial respiration, (**C**) maximal O_2_ consumption and (**D**) ATP production values result from the subtraction of each parameter with the first value of OCR after rotenone+antimycin injection. Data are the mean ± SEM expressed in absolute values of five different control individuals and three BD patients after the correction with non-mitochondrial OCR provided by the injection of rotenone+antimycin. (**E**,**F**) Glycolytic capacity was assessed by sequential addition of oligomycin and 2-deoxyglucose (2-DG). (**E**) Representative traces and (**F**) glycolytic capacity. Data are the mean ± SEM expressed in absolute values of five different control individuals and 3 BD patients after the correction with non-glycolytic ECAR provided by the injection of 2-deoxyglucose. Experiments were carried out in duplicate. * *p* < 0.05; ** *p* < 0.01, significantly different from control group, as determined by Mann–Whitney non-parametric test.

We next evaluated whether the observed alterations in mitochondrial morphology, dynamics and mitochondrial membrane potential were related to overall impaired metabolic function. We studied cellular bioenergetics following mitochondrial metabolic stress using a Seahorse XF24 flux analyzer. OCR measurements were taken at basal levels and following sequential addition of mitochondrial respiration inhibitors: oligomycin, carbonyl cyanide-p-trifluoromethoxyphenylhydrazone (FCCP), and a combination of antimycin A and rotenone. Real-time measurements of OCR are shown in Figure 5A, and the quantitation of results is shown in Figure 5B–D.

The results showed decreased maximal oxygen consumption as well as decreased ATP production in mitochondria from BD patients’ fibroblasts in comparison with controls. These results suggest that mitochondria from BD fibroblasts have lower respiratory capacity than mitochondria from control fibroblasts.

Moreover, glycolytic capacity, which indicates energy production that is independent of mitochondrial respiration, was performed by measuring the ECAR of BD patients and control fibroblasts following a sequential addition of glucose, oligomycin, and 2-deoxyglucose (2-DG; Figure 5E,F). BD fibroblasts have a higher glycolytic capacity when compared to the controls, which indicates higher energy production that is independent of mitochondrial respiration.

## 4. Discussion

A significant objection to studying BD is the limited access to viable central nervous system (CNS) tissue and thus cell-based models should significantly impact our understanding of BD genesis [36]. Patient-derived dermal fibroblast culture is a promising *in vitro* model to study cellular, molecular, metabolic, and pathophysiological features of psychiatric disorders [37]. Dermal fibroblasts are an accessible source of patient cells, are easy to establish and maintain in culture without transformation, and the majority of confounding factors (e.g., life style or medication use) are practically eliminated after several rounds of cell division. Hence, in this study, primary dermal fibroblasts have been used as an extraneural model to evaluate mitochondria alterations during the initial stages of BD.

Mitochondrial dysfunction has been associated with psychiatric disorders such as BD [8,38]. Evidence of mitochondrial alterations has been reported in BD patients [18,21,22,39] and, in turn, a high prevalence of affective syndromes, particularly BD, have been shown in adults with primary mitochondrial diseases [12]. This study provides evidence supporting that alterations in mitochondria occur in early phases of BD and that is evident in non-neuronal peripheral cells. Our results, which were obtained in primary fibroblasts, show that mitochondrial morphological alterations (fragmentation) and unbalanced fusion/fission processes are features of the initial stages of BD. Mitochondrial dysfunction arising from inhibition of the activity of the electron transport chain leading to ATP depletion was also a feature of BD fibroblasts. Mitochondria fragmentation seems to be compensated with increased mitochondrial biogenesis and elimination of dysfunctional mitochondria by macroautophagy (mitophagy), as well as increased glycolytic capacity.

We found that mitochondrial morphology in BD fibroblasts is altered when compared with control fibroblasts, as demonstrated by increased roundness and decreased length (aspect ratio). Our results are in line with a postmortem study reporting that neuronal cells in the prefrontal cortices of patients with BD, but also peripheral cells from living BD patients, display abnormalities in the morphology and intracellular distribution of mitochondria, which are more abundant and have reduced size [20]. A more recent study using iPSC-derived fibroblasts also showed smaller mitochondria in BD patients in comparison with controls [21].

Mitochondria are highly dynamic organelles that constantly fuse and divide, and it is known that the balance between these two processes (fission and fusion) regulates mitochondrial network morphology. An imbalance between fission and fusion processes can compromise cell function, leading to cell death. In fact, we observed changes in the levels of markers of fusion and fission events, which might explain alterations in mitochondrial morphology that we observed in BD fibroblasts. This imbalance in mitochondrial dynamics is in agreement with a study that reported alterations in fission/fusion in BD, where it was observed downregulation of the mitochondrial fusion-related proteins Mfn-2 and Opa-1 and the upregulation of the fission protein Fis-1 in PBMCs from patients with BD [22]. We also showed a tendency to an increase in the levels of mtTFA, a biogenesis marker, in BD fibroblasts. Elevated biogenesis markers can translate into increased mitochondria, a possible compensation mechanism, where the cell responds in order to restore normal mitochondrial function. Results from available literature are controversial concerning biogenesis of mitochondria in BD. Some studies described reductions, while others showed no alterations in mitochondrial DNA copy number in peripheral blood cells of BD patients [22,40,41,42,43,44]. Other studies showed increased mitochondrial DNA copy number in blood cells and in the postmortem brain of BD patients [45,46], which is in accordance with our findings. Our study showed that fibroblasts from BD patients exhibited decreased MMP in comparison with control fibroblasts, supporting mitochondrial dysfunction in BD. However, it has been reported that neurons derived from iPSCs of BD patients had increased MMP [21]. Mitochondrial depolarization has been considered as the main event for activating mitophagy machinery, which is important to maintain cell homeostasis by degrading damaged mitochondria [10]. Our results suggest increased mitophagy in BD, since increased mitochondria-autophagosome co-localization was detected in BD fibroblasts, together with evidence of stimulation of autophagosome formation and lysosomal degradation. In contrast, it was observed that mitophagy-related proteins were downregulated in PBMCs of BD patients supporting the conclusion that the number of damaged mitochondria exceeds the capacity of mitophagy in the disease [23]. In our study, increased mitophagy in fibroblasts from early BD patients, together with increased mitochondrial biogenesis, can represent a mechanism to preserve mitochondrial structure and function and maintain cell homeostasis and survival.

Several studies show that mitochondrial morphology is linked to alterations in energy metabolism [47,48]. The present study shows a decrease in maximal O_2_ consumption and ATP levels and increased glycolytic capacity in BD patients-derived fibroblasts. When mitochondrial function is inhibited, glycolytic ATP production increases as a result of impaired oxidative phosphorylation. However, glycolysis is less efficient than mitochondrial respiration [49]. Our results are in agreement with several previous reports. Decreased expression and activity of the ETC components, namely complex I have been described in postmortem frontal cortex of BD patients [16,18,50]. Moreover, decreased respiration after complex I inhibition and increased residual respiration were found in blood platelets from depressive BD patients compared to controls [39]. Another study reported increased lactate in the cerebrospinal fluid of BD patients, indicating decreased mitochondrial respiration [51]. Moreover, PBMCs from control subjects showed upregulated expression of ETC genes in response to glucose deprivation, while cells from BD patients did not [52]. Together, these studies support our results and suggest that impaired ETC within the mitochondria may be associated with a shift from oxidative phosphorylation to anaerobic glycolysis in BD. Thirty-one P-MRS studies in adolescents showing that inorganic phosphate was decreased in medication-free patients compared to medicated patients and controls [53] also highlight the low energy status of BD patients.

Mitochondrial biogenesis is a compensatory response to increased energy requirement. Since we observed increased mitochondrial biogenesis markers in BD patients-derived fibroblasts we would expect to observe an increase in the production of ATP instead of a decrease. According to our results, the increase in mitochondrial biogenesis seems to be insufficient to compensate the energetic deficit. It is tempting to speculate that the new synthesized mitochondria have decreased expression and/or activity of the ETC components [16,18,50], which could explain why the increased mitochondrial biogenesis does not translate into increased ATP production.

In summary, we observed alterations in mitochondrial morphology, unbalance between mitochondrial fission and fusion events and increased biogenesis in fibroblasts from BD patients versus controls. The decrease in the MMP, the enhanced co-localization between mitochondria and autophagosome markers, as well as the decreased p62 levels, which occurred in a reduced extent, suggest activation of macroautophagy, in particular an increase in the mitophagy process. Under these conditions, inhibition of mitochondrial respiration and ATP depletion were observed in BD fibroblasts. In response to the first changes in mitochondria function during early stages of the disease, mild to moderate alterations in mitochondrial dynamics, biogenesis and mitophagy, together with glycolysis stimulation, might represent an adaptive strategy to maintain cell homeostasis. It would be interesting to also assess mitochondrial alterations during later stages of this pathology. Nevertheless, it is worth noting that this study is a proof of concept that should be further explored with a higher number of participants.

This study, performed using a patient-derived cell model, provides relevant information supporting that mitochondrial dysfunction occurs in early phases of BD and can play a crucial role in the development and progression of the disease.

## Data Availability

The data presented in this study are openly available in https://drive.google.com/drive/folders/1X7lAouCiB-femCTygb_pnpioIvFfnOGQ?usp=sharing (accessed on 1 May 2021).
